# Supercritical CO_2_ Extraction of Bioactive Compounds from *Vitis labrusca* Grape Marc: Effects of Operating Conditions and Pilot-Scale Validation

**DOI:** 10.3390/molecules31132272

**Published:** 2026-06-29

**Authors:** Camilo Pardo-Castaño, Alejandro Quintero-Velez, William Fernando Vallejo-Revelo

**Affiliations:** 1 School of Chemical Engineering, Universidad del Valle, Calle 13 No. 100-00, Cali 760032, Colombia; 2 Didacontrol S.A.S., Calle 32 No. 1-22, Cali 760001, Colombia

**Keywords:** supercritical CO_2_ extraction, bioactive compounds, *Vitis labrusca*, grape marc, phenolic compounds, antioxidant capacity, design of experiments, scale-up

## Abstract

Grape marc (*Vitis labrusca*), a major by-product of the winemaking industry, is generated in large quantities and represents a promising source of bioactive compounds. This residue is particularly rich in phenolic metabolites associated with antioxidant activity. In this study, supercritical CO_2_ extraction was investigated as a sustainable strategy for the recovery of bioactive compounds from *Vitis labrusca* grape marc. A 2^4−1^ fractional factorial design was employed to evaluate the effects of temperature (30–60 °C), pressure (137.9–275.8 bar), ethanol concentration (0–10 wt%), and particle size (116–601 µm) on extraction yield, total phenolic content (TPC), and antioxidant capacity (AC). Extraction performance was strongly influenced by operating conditions, revealing a clear trade-off between recovery and selectivity. The highest extraction yield (8.3 wt%) was obtained using 10 wt% ethanol as co-solvent, whereas the highest antioxidant capacity (365.3 µmol TE/g extract) was achieved under neat CO_2_ conditions. TPC values reached approximately 69 mg GAE/g extract and were significantly affected by the combined effects of temperature, particle size, and ethanol concentration. The results revealed two distinct extraction regimes: a high-recovery regime promoted by ethanol addition and a high-selectivity regime under neat CO_2_ conditions. Representative extracts were further characterized by UHPLC-QTOF-MS/MS. Ethanol-modified extraction was associated with higher relative abundance and diversity of flavonoids, stilbenes, and phenolic acids, whereas neat CO_2_ extraction favored lipophilic metabolites such as oxylipins and unsaturated fatty acids. Selected operating conditions were successfully reproduced at pilot scale, supporting the scalability of the process. Overall, the results demonstrate that supercritical CO_2_ extraction can be tailored to recover bioactive compounds from grape marc as extracts with distinct chemical profiles and provide a viable strategy for the valorization of *Vitis labrusca* winemaking residues.

## 1. Introduction

Grape production is one of the largest agricultural sectors worldwide and generates significant amounts of agro-industrial residues during winemaking. According to the International Organization of Vine and Wine (OIV, 2024) [1], global grape production reaches 77.7 million tons, of which 47% is used for wine production, while the remainder is allocated to fresh consumption (46%) and dried products (7%). The winemaking process produces a wide variety of residues, typically characterized by the presence of biodegradable compounds and suspended solids. These materials are particularly rich in bioactive secondary metabolites, especially phenolic compounds.

In recent years, grape by-products have attracted increasing attention as a potential source of high added-value materials, mainly due to their antioxidant [2,3] and antimicrobial activities [4]. At the same time, their disposal may lead to environmental concerns such as water pollution, soil degradation, damage to vegetation, energy consumption, and the emission of gases and odors. For this reason, there is a growing interest in developing more sustainable strategies for their valorization, in line with current global efforts toward sustainability [5,6,7].

The main by-product of the winemaking industry is grape marc, a mixture of grape skins, seeds, and stalks. It is estimated that approximately 1 kg of grape marc is generated for every 5 L of wine produced, corresponding to about 20–30% of the total grape weight [6,8]. Owing to its composition, this residue can be transformed into a wide range of products of industrial interest, including food additives, nutraceuticals, dietary supplements, pharmaceuticals, fertilizers, animal feed, antimicrobial agents, cosmetics, and biomass for biofuels [8,9].

The composition of grape marc is complex and depends on environmental factors (e.g., soil and climate) as well as viticultural conditions (e.g., grape variety, fertilization, and harvest time). Red grape marc generally contains higher levels of polyphenols than white grape marc. In this case, the skins account for 52–56% of the dry matter and contain the highest concentration of polyphenols, particularly anthocyanins, stilbenes, and tannins. The seeds represent 38–52% on a dry basis (DB) and are rich in fiber (40 wt%), proteins (11 wt%), lipids (16 wt%), and polyphenols (7 wt%), among other compounds. In contrast, the stalks account for approximately 14% of the total residue and are mainly composed of lignin, cellulose, hemicellulose, ash, and polyphenols (6 wt% DB) [7].

While grape pomace from *Vitis vinifera* has been extensively investigated, considerably less information is available for *Vitis labrusca* residues despite their importance in several South American wine-producing regions. Previous studies have shown that *Vitis labrusca* pomace, particularly from Isabella cultivars, contains a wide variety of phenolic and lipophilic compounds, including anthocyanins, flavonoids, stilbenes, phenolic acids, and seed-derived bioactive constituents [10,11,12]. These compositional differences may influence extraction behavior and extract composition when supercritical fluids are employed. Consequently, results obtained for *Vitis vinifera* cannot necessarily be extrapolated to *Vitis labrusca* residues.

The extraction of phenolic compounds from plant materials is a key step in their valorization, and several techniques have been explored for grape marc. These include ethanol–water extraction [13], supercritical fluid extraction [11,12,14], and pressurized liquid extraction [15]. Among these alternatives, supercritical CO_2_ extraction (SFE) has attracted considerable attention as a green extraction technology. One of its main advantages lies in the properties of CO_2_, which is inert, non-toxic, and environmentally safe, while also allowing operation under relatively mild temperature conditions. In addition, SFE often leads to extracts of higher purity compared to those obtained using conventional organic solvents, and these extracts are generally recognized as safe (GRAS) for applications in food, cosmetic, and pharmaceutical products [16].

Despite the growing interest in grape pomace valorization, information regarding *Vitis labrusca* residues remains comparatively scarce. Most available studies have focused either on extraction yield, phenolic recovery, or antioxidant activity and have generally been restricted to laboratory-scale assessments. To the best of our knowledge, no previous study has integrated process optimization, pilot-scale validation, and molecular-level characterization to investigate how operating conditions affect the recovery and selectivity of supercritical CO_2_ extracts from *Vitis labrusca* grape marc. Such an integrated approach is necessary to understand the mechanisms governing extraction performance, establish the relationship between process conditions and extract composition, and support the industrial implementation of supercritical fluid extraction as a valorization strategy for winery residues.

Therefore, the objective of this work was to evaluate the effects of temperature, pressure, ethanol concentration, and particle size on the supercritical CO_2_ extraction of bioactive compounds from *Vitis labrusca* grape marc using a fractional factorial design. Extraction yield, total phenolic content, and antioxidant capacity were employed as response variables to investigate the balance between extraction efficiency and selectivity. The extraction conditions identified at laboratory scale were subsequently assessed in a pilot-scale unit to evaluate process scalability. Additionally, representative extracts obtained under neat CO_2_ and ethanol-modified CO_2_ conditions were characterized using UHPLC-QTOF-MS/MS-based untargeted profiling, providing further insight into the phenolic and lipophilic compounds associated with each extraction regime.

## 2. Results

### 2.1. Experimental

Table 1 summarizes the results of the 2^4−1^ fractional factorial design proposed in this study. The responses were evaluated after 90 min of extraction and include extraction yield (%Y), total phenolic content (TPC, expressed as mg GAE/g extract), and antioxidant capacity (AC, expressed as µmol TE/g extract). The experimental factors considered were temperature (T), pressure (P), ethanol concentration as co-solvent (C), and average particle size (S). These factors are presented as coded variables ranging from −1 to 1. Central points (0, 0, 0, 0) were included to evaluate curvature and experimental reproducibility.

**Figure 1:**
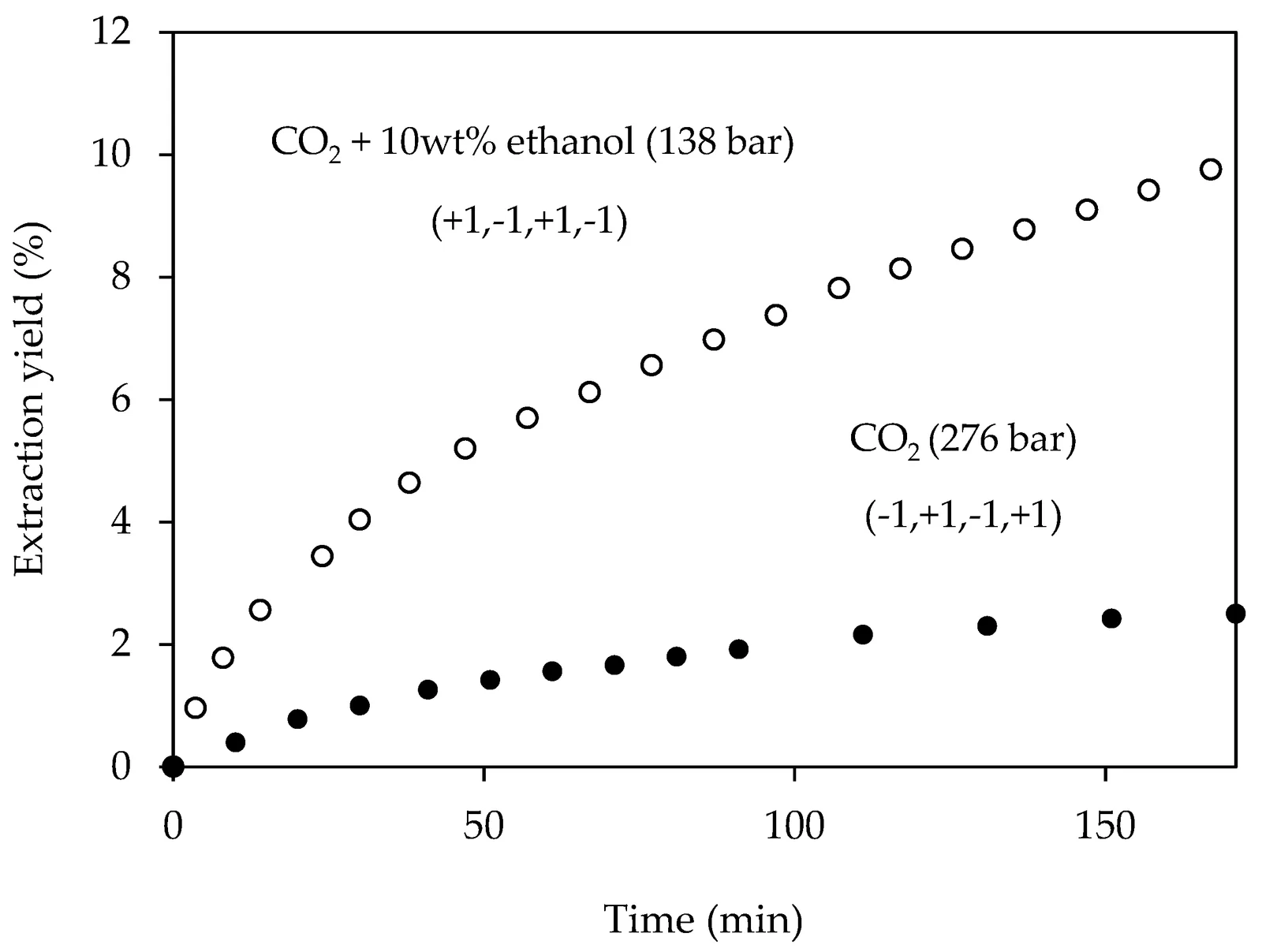
Comparison of extraction kinetics under neat CO_2_ and ethanol-modified CO_2_ conditions. The curves show the evolution of cumulative extraction yield with extraction time and highlight the influence of ethanol on extraction efficiency.

To further illustrate the differences between extraction regimes, the extraction kinetics corresponding to the best experimental runs obtained with neat CO_2_ and with co-solvent addition are presented in Figure 1. The curves show clear differences between the two systems. Neat CO_2_ leads to a lower overall extraction yield, although it tends to favor the selective recovery of compounds with high antioxidant activity. The addition of ethanol produces a different response, increasing extraction yield while allowing the recovery of a broader range of bioactive compounds. Differences are also observed in the kinetic profiles, particularly during the initial stages of extraction, where faster extraction rates are obtained in the presence of co-solvent. This behavior can be associated with improved solubility and enhanced desorption of more polar compounds from the matrix. Taken together, these observations point to a trade-off between selectivity and extraction efficiency, highlighting the role of solvent polarity in controlling both extraction kinetics and final extract composition. Additional kinetic profiles confirmed the effect of pressure and solvent composition on extraction performance (see Supplementary Figure S2).

The experimental results showed a broad dispersion across all response variables, which reflects the sensitivity of the system to changes in operating conditions. Extraction yield ranged from 0.3 to 7.1%, while total phenolic content varied between 6.3 and 69.3 mg GAE/g, and antioxidant capacity spanned from 40.8 to 365.3 µmol TE/g. The highest yield (7.1%) was obtained under conditions combining high temperature, low pressure, the presence of ethanol, and a smaller particle size. This combination appears to favor both mass transfer and solubilization, particularly for compounds with higher polarity.

In contrast, the highest antioxidant capacity and total phenolic content were observed under neat CO_2_ conditions, at high pressure and larger particle size. Even though these conditions led to lower yields, they point to a more selective extraction regime, where compounds with stronger antioxidant activity are preferentially recovered. This observation highlights the complex relationship between extraction yield and extract composition, emphasizing the need to balance both quantity and quality during process optimization.

The central points included in the experimental design showed a moderate variability in extraction yield, which is consistent with an acceptable level of experimental reproducibility under the selected operating conditions. This behavior gives confidence in the consistency of the experimental data, although some dispersion is still expected in this type of system.

From a general perspective, these observations suggest that the process does not respond uniformly to the operating variables. Instead, different mechanisms seem to dominate depending on the conditions, which makes it necessary to examine the data from a statistical perspective to separate individual effects from interaction-driven behavior.

### 2.2. Significance of Factors

The experimental results were analyzed using analysis of variance (ANOVA) together with the statistical models obtained from the fractional factorial design. The statistical analyses were performed using JMP Pro (Version 14, SAS Institute Inc., Cary, NC, USA). Table 2 summarizes the *p*-values associated with the main effects and selected binary interactions of the studied factors. It should be noted that, due to the fractional nature of the 24 − 1 design, it is not possible to independently estimate all interaction effects, since some of them are confounded. Consequently, higher-order interactions (ternary and quaternary) cannot be resolved within this experimental design.

The initial analysis of variance (ANOVA) showed that all the investigated factors, together with the selected interactions, had a statistically significant effect on the response variables at a 95% confidence level (*p* < 0.05). Among these, the interactions C × T and C × S, as well as the main effects of S and T, stood out with the highest statistical significance (*p* < 0.01), pointing to their dominant influence within the system. Pressure (P), ethanol concentration (C), and the interaction C × P were also identified as significant, although their contribution was comparatively less pronounced.

Rather than acting independently, several variables appear to influence the system through their combined effects. This is especially evident in the interactions involving ethanol concentration, which modifies how the system responds to changes in temperature and particle size. In this sense, ethanol behaves less as a primary driver and more as a factor that alters the extraction environment.

From these results, it becomes evident that ethanol concentration plays an important role in the extraction process. Its effect is not limited to acting as an independent variable but is mainly expressed through its interactions with other operating factors. In particular, the strong contribution of C × T and C × S indicates that both temperature and particle size are closely linked to the solvent composition, reflecting the coupled behavior of the system.

Although the initial ANOVA indicated statistical significance for several terms, a closer inspection of the model through residual analysis revealed some inconsistencies with the assumptions of normality and homoscedasticity when all terms were retained. These deviations were mainly observed around the central region of the experimental domain, where the residuals showed a non-random pattern. This type of behavior is often associated with the presence of curvature, suggesting that a purely linear model may not be sufficient to fully capture the relationships between the variables.

In systems such as this, where temperature, solvent composition, and matrix characteristics interact simultaneously, some degree of curvature is not unexpected. While the inclusion of central points made it possible to detect this effect, the fractional factorial design used here does not allow the independent estimation of quadratic terms.

Given these limitations, the model was refined with a stronger emphasis on residual behavior and overall adequacy rather than strictly following statistical significance. As part of this step, the main effect of ethanol concentration (C) and its interaction with pressure (C × P) were excluded. Their contribution was comparatively small, and their removal led to a noticeable improvement in the distribution of residuals.

It is worth noting that this adjustment departs from the hierarchy principle. Even so, the resulting model showed a more consistent behavior, with residuals randomly distributed and no evident systematic trends. Under these conditions, the model can be considered a more reliable representation of the system within the studied domain.

The influence of the operating variables after this refinement is summarized in Table 3. Considering both statistical significance and the relative contribution of each factor provides a clearer view of how the system behaves.

Table 3 highlights that factor S and temperature (T) are the variables with the strongest overall influence on the extraction process. Both show clear direct effects and participate in the most relevant interaction terms, which explains their dominant role within the system. In comparison, pressure (P) has a more limited influence. Although it appeared statistically significant in the initial ANOVA, its contribution becomes less relevant after model refinement, suggesting a secondary role within the studied experimental domain.

Ethanol concentration (C) behaves somewhat differently. Even though its main effect was removed during model refinement, its overall influence remains evident through its participation in significant interaction terms, particularly C × T and C × S. This suggests that ethanol does not act as an independent variable, but rather modifies the effect of temperature and S.

From this perspective, the extraction process can be understood as being mainly governed by the combined effects of temperature and S. Under these conditions, ethanol plays an indirect but still important role through interaction mechanisms, while the contribution of pressure remains limited.

### 2.3. Data Correlation

The relationship between the independent variables and the response variables was described using a second-order polynomial regression model, as presented in Equation (1). Quadratic terms were not included due to the fractional nature of the experimental design, which limits the independent estimation of second-order effects.

(1) $$Y=\beta_{0}+\sum_{i\;=1}^{4}\beta_{i}X_{i}+\sum_{i\;\text{<}\;j}^{4}\beta_{\mathrm{ij}}X_{i}X_{j}$$

where Y represents the predicted response (extraction yield, total polyphenol content, or antioxidant capacity), β_0_ is the intercept, β_i_ are the linear coefficients, and β_ij_ correspond to the interaction coefficients between variables. The coded variables X_1_, X_2_, X_3_, and X_4_ correspond to temperature, pressure, ethanol concentration, and particle size, respectively.

The estimated regression coefficients for total phenolic content (TPC), antioxidant capacity (AC), and extraction yield (Y) are reported in Table 4. In the case of extraction yield, a natural logarithmic transformation (lnY) was applied to improve compliance with the regression assumptions. The statistical analysis showed that the relevance of the factors depends on the response variable considered, which reflects the different mechanisms governing each response within the extraction process.

The resulting empirical models for each response variable can be expressed, in terms of coded variables, as follows:

Total phenolic content (TPC):

TPC = 39.52 + 10.63S + 9.28T − 10.70(C × S) + 11.70(C × T)(2)

Antioxidant capacity (AC):

AC = 178.45 + 46.64S + 36.04P − 54.54(C × S) + 37.64(C × T)(3)

Extraction yield (ln(Y)):

ln(Y) = 0.830 + 0.775(T)(4)

For TPC, both factor S and temperature (T) showed significant positive effects (*p* < 0.05), so increasing these variables generally leads to a higher recovery of phenolic compounds within the studied experimental domain. In contrast, pressure (P) did not show a statistically significant effect within the evaluated range. The interaction terms involving ethanol concentration (C) were also significant, with C × S contributing negatively and C × T positively. This behavior indicates that ethanol does not operate as an independent factor, but instead modifies the system through its interactions with the other variables. Similar observations have been reported in previous studies, where cosolvents alter the polarity of the extraction medium, affecting both solubility and selectivity of phenolic compounds [16].

A similar tendency was observed for antioxidant capacity (AC), where factor S remained significant, confirming its influence on extract quality. The interaction terms C × S and C × T were again relevant, which further supports the idea that ethanol mainly acts through interaction effects. Pressure showed a marginal contribution (*p* = 0.0569), possibly related to changes in solvent density and solvation behavior. Although the statistical significance of pressure was slightly above the conventional threshold (*p* = 0.0569), this term was retained in the final AC model because its inclusion improved residual diagnostics and overall model adequacy. Therefore, model selection was based not only on statistical significance but also on predictive performance and consistency with the underlying extraction phenomena. In contrast, temperature did not present a statistically significant main effect for AC, which suggests that its influence is largely expressed through interactions rather than as an independent variable.

In contrast to TPC and AC, the extraction yield (Y) followed a different pattern. Temperature was the only statistically significant factor (*p* < 0.05), while the remaining terms were not significant within the studied range. This points to a process mainly governed by temperature-dependent phenomena, such as enhanced mass transfer and increased solubility of compounds, which are commonly observed in extraction processes involving natural matrices [17].

### 2.4. Effect of Interactions and Role of Ethanol

The interaction effects involving ethanol concentration (C) offer a more detailed view of how the extraction system behaves. The negative coefficient associated with C × S indicates that increasing ethanol content may reduce extraction selectivity, which could be related to the co-extraction of non-phenolic compounds or the presence of matrix interferences. Similar behavior has been reported in previous studies, where the addition of cosolvents increases solvent polarity and favors the extraction of a broader range of compounds [16,18].

In contrast, the positive interaction between C and T points to a different type of effect. Under these conditions, higher temperatures appear to enhance the role of ethanol as a cosolvent, leading to improved recovery of phenolic compounds. This combined effect has been observed in other extraction systems, where temperature facilitates mass transfer and solvent penetration, while cosolvents strengthen solute–solvent interactions [17,18].

Overall, ethanol does not behave as an independent variable but rather as a factor whose influence depends strongly on the operating conditions. Its effect is closely linked to temperature and to the characteristics represented by factor S, which reflects the inherent complexity of the extraction process.

### 2.5. Comparative Analysis of Responses

When the three responses are examined together, a clear difference emerges between extraction yield and extract quality (TPC and AC). While TPC and AC are strongly influenced by interaction effects and more selective mechanisms, extraction yield is mainly governed by temperature and reflects overall solubilization rather than selectivity. Similar trends have been reported in the extraction of natural matrices, where yield is typically associated with mass transfer and solubility, whereas the recovery of bioactive compounds depends more on selective interactions between solvent composition and matrix characteristics [15,17].

These observations point to the coexistence of different mechanisms within the extraction process. Increasing extraction yield does not necessarily lead to an improvement in extract quality, since the variables that favor mass transfer and solubility are not the same as those that promote the selective recovery of phenolic compounds. This balance between extraction efficiency and selectivity has been widely discussed in studies dealing with plant-derived bioactive compounds [15,18].

### 2.6. Model Refinement and Adequacy

The main effect of ethanol concentration (C), together with its interaction with pressure (C × P), was ultimately removed from the model, as both terms showed a limited contribution, and their inclusion led to less consistent residual behavior. Although this type of adjustment does not strictly comply with the hierarchy principle, it resulted in a model that better reflects the experimental data, particularly in terms of residual distribution, which became more random and freer of evident systematic patterns.

Model performance was then evaluated in more detail through residual diagnostics for all response variables (TPC, AC, and Y). When examining the residuals as a function of the predicted values, no clear structure or trend could be identified, while the normal probability plots showed an acceptable approximation to normality (see Supplementary Figure S1). Taken together, these observations support the use of the refined models to describe the influence of the operating variables within the studied experimental domain.

The absence of a significant lack of fit (*p* > 0.05) further supports the adequacy of the final models. Even so, the predictive capability remains somewhat limited for certain responses, which may be related to effects not fully captured by the fractional factorial design. In particular, the presence of curvature or inherent variability in the system cannot be ruled out. This interpretation is consistent with the behavior observed in the residual analysis and suggests that higher-order effects could play a role under the studied conditions.

### 2.7. Overall Interpretation

The results point to an extraction process governed by both general solubilization phenomena and more selective interaction mechanisms. In this context, temperature appears as the main variable controlling extraction yield, while total phenolic content and antioxidant capacity are more closely linked to interaction effects, particularly those associated with solvent composition and operating conditions.

From this perspective, the system reflects the coexistence of different mechanisms that do not necessarily evolve in the same direction. Increasing extraction yield does not automatically translate into better extract quality, since the variables that enhance mass transfer differ from those that favor the selective recovery of bioactive compounds. This highlights the need to approach process optimization in a balanced way when both efficiency and extract quality are relevant.

### 2.8. Optimization

**Figure 2:**
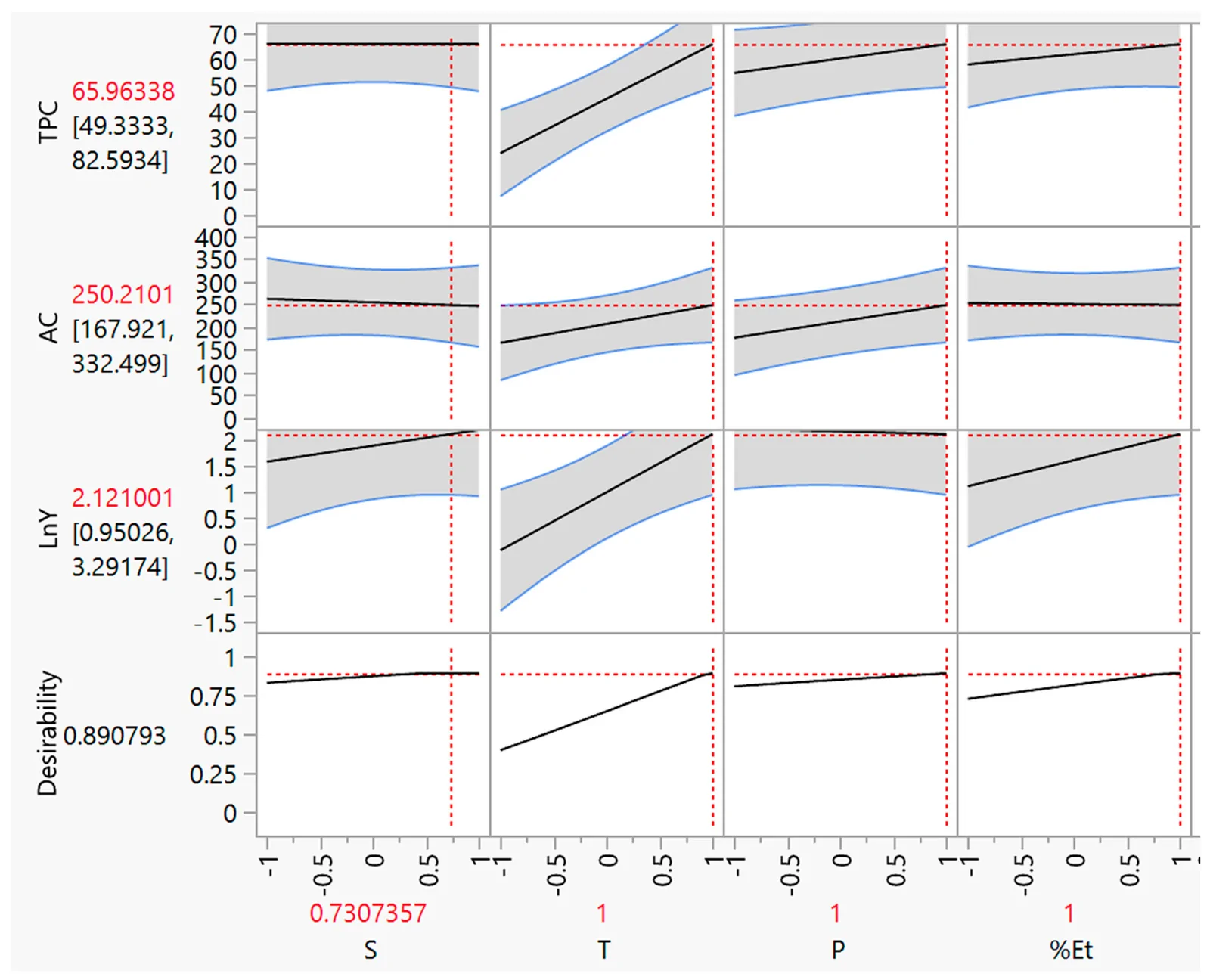
Prediction profiler showing the effects of particle size (S), temperature (T), pressure (P) and ethanol concentration (%Et) on extraction yield (LnY), antioxidant capacity (AC) and total phenolic content (TPC). Shaded areas represent the 95% confidence intervals, and red dashed lines indicate the evaluated operating conditions.

Figure 2 shows the prediction profiler used to explore how the selected factors can be adjusted to simultaneously improve the three response variables within the studied experimental domain. The optimization was carried out using a desirability function, in which the individual models for each response are combined into a single objective.

According to the model predictions, the optimal conditions are associated with the largest particle size (601 µm), the highest temperature (60 °C), the highest pressure (275.8 bar), and the highest ethanol concentration (10%) within the evaluated range. Under these conditions, the predicted responses correspond to a yield of 8.3 ± 3.2%, a total polyphenol content (TPC) of 66.0 ± 16.6 mg GAE/g, and an antioxidant capacity (AC) of 250.2 ± 37.3 µmol TE/g, with an overall desirability of 0.89.

Within the experimental uncertainty, these conditions match the last experimental run listed in Table 1. The measured values (5.2 ± 0.9% yield, 65.6 ± 0.9 mg GAE/g TPC, and 244.6 ± 2.9 µmol TE/g AC) are in close agreement with the predicted ones, which gives confidence in the consistency of the optimization procedure.

Model performance was also examined using the coefficients of determination (R^2^) and adjusted coefficients (R^2^adj), which were 0.92 and 0.83 for TPC, 0.88 and 0.76 for AC, and 0.78 and 0.56 for yield, respectively. While the models for TPC and AC show a good capacity to explain the observed behavior, the lower R^2^adj value for yield points to a more limited predictive performance for this response and suggests that its interpretation should be handled with care. For this reason, particular attention was given to the residual analysis, since goodness-of-fit metrics alone may not fully reflect model adequacy. Residuals were therefore examined in terms of randomness, normality, and homoscedasticity, in order to rule out systematic deviations associated with model misspecification. Based on this combined assessment, the models provide a reasonable description of the system, especially for TPC and AC.

**Figure 3:**
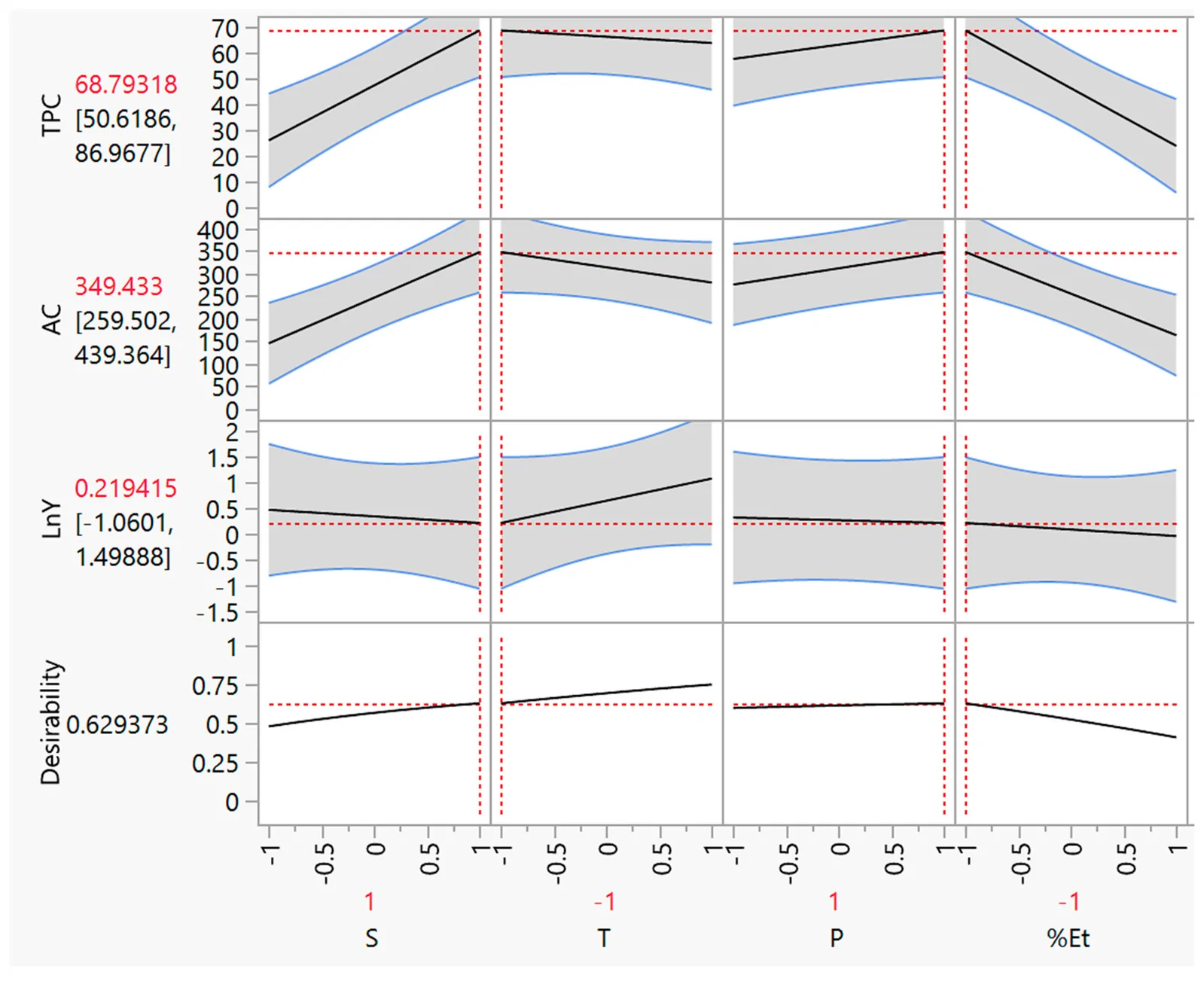
Prediction profiler showing the effects of particle size (S), temperature (T), pressure (P) and ethanol concentration (%Et) on extraction yield (LnY), antioxidant capacity (AC) and total phenolic content (TPC) under neat CO_2_ extraction conditions. Shaded areas represent the 95% confidence intervals, and red dashed lines indicate the evaluated operating conditions.

An additional observation arises from both the experimental data and the prediction profiler (see Table 1 and Figure 3). It is possible to obtain extracts with high antioxidant potential using neat CO_2_, without the need for a co-solvent. One of the experimental runs confirmed that, at 275.8 bar, 30 °C, and a particle size of 601 µm, extracts with high total polyphenol content (≈69.3 mg GAE/g) and the highest antioxidant capacity (≈365.3 µmol TE/g) can be obtained. This result is consistent with the model predictions and supports the existence of a high-selectivity extraction regime under neat CO_2_ conditions.

The improvement in extract quality comes at the expense of a noticeable reduction in extraction yield (≈2%). This can be linked to the limited solubility of polar compounds in non-modified supercritical CO_2_. Under these conditions, CO_2_ shows a clear preference for less polar species, so only a fraction of the phenolic compounds is effectively recovered. When ethanol is introduced as a co-solvent, the behavior changes, as the polarity of the supercritical phase increases, which facilitates the solubilization and recovery of a broader range of phenolic compounds and leads to higher overall yields. In this sense, the system exhibits a balance between selectivity and efficiency: neat CO_2_ tends to produce extracts with higher activity, while the presence of a co-solvent favors total recovery.

To explore whether the low yields obtained under neat CO_2_ conditions could be improved, additional experiments were carried out at higher pressures. At 30 °C and 400 bar, a yield of 3.6% was obtained after 40 min, increasing to 4.0% at 500 bar under the same conditions. This increase is consistent with the higher density and solvent strength of supercritical CO_2_ at elevated pressures. Even so, the yields remain below those achieved with co-solvent addition and longer extraction times (90 min in the experimental design). This suggests that, although pressure contributes to improving solute solubility, it does not fully compensate for the absence of a co-solvent. In practice, the low polarity of CO_2_ still limits the overall recovery of many phenolic compounds. These observations reinforce the idea that solvent polarity plays a more decisive role than density alone when targeting polar bioactive compounds.

### 2.9. Pilot-Scale Validation of Extraction Conditions

**Figure 4:**
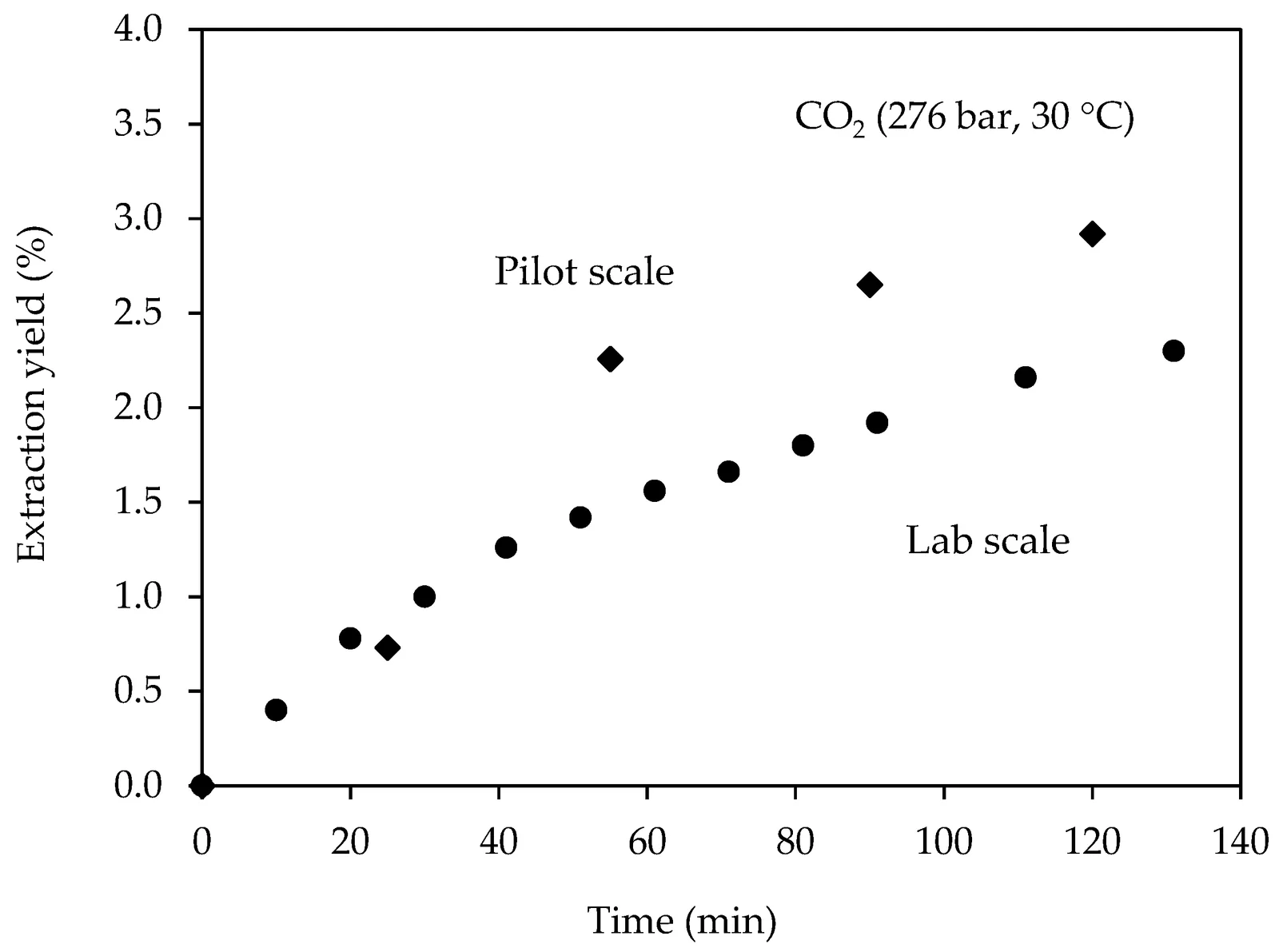
Comparison of extraction kinetics at laboratory (30 °C, 276 bar, 601 µm, S/F = 0.30 min^−1^) and pilot scales (30 °C, 276 bar, 770 µm, S/F = 0.36 min^−1^), illustrating process scalability under similar operating conditions. Diamond symbols represent pilot-scale experiments, whereas circles correspond to laboratory-scale experiments.

The scalability and practical applicability of the process were assessed through pilot-scale extraction experiments carried out under conditions comparable to those identified at laboratory scale for neat CO_2_ extraction. Due to scale-up limitations, the average particle size was increased from 601 to 770 µm, while the solvent-to-feed ratio was adjusted from 0.30 to 0.36 min^−1^. Under these conditions, and operating at 30 °C and 276 bar, a yield of 2.9 wt% was obtained after 2 h. The corresponding extraction kinetics are shown in Figure 4.

In principle, an increase in particle size would be expected to reduce extraction efficiency. In this case, however, the higher solvent-to-feed ratio provided greater solvent availability, which appears to have favored solute removal. In addition, some improvements in hydrodynamic behavior at pilot scale cannot be ruled out. Factors such as a more uniform solvent distribution along the extraction bed and a reduction in channeling may have contributed to the slightly higher yield compared to laboratory-scale runs under neat CO_2_ conditions.

Overall, the same extraction trend observed at the laboratory scale is maintained after scale-up. At the same time, the results suggest that moderate adjustments in operating parameters can partially offset the geometric and mass transfer limitations typically associated with larger-scale systems.

### 2.10. Chemical Profiling of Representative Extracts by UHPLC-QTOF-MS/MS

To gain further insight into extraction selectivity, representative extracts obtained under the optimal conditions identified for neat CO_2_ and ethanol-modified CO_2_ extraction were analyzed by UHPLC-QTOF-MS/MS. Compound annotation was performed based on accurate mass measurements, isotopic patterns, fragmentation spectra, and database matching. An initial set of annotated features was obtained and subsequently subjected to manual curation to retain only chemically plausible metabolites relevant to grape-derived matrices and the objectives of this study. This procedure resulted in a curated dataset comprising 79 tentatively annotated compounds, which was used for subsequent interpretation and comparison of the extracts. The complete list of curated compounds is provided in Supplementary Table S1.

The curated compounds were grouped into lipophilic and phenolic classes to facilitate interpretation of extraction selectivity. Lipophilic metabolites were mainly represented by oxylipins, unsaturated fatty acids, monoacylglycerols, dicarboxylic acids, sphingolipid-related compounds, and terpenoids, whereas the phenolic fraction comprised flavonoids, stilbenes, phenolic acids, coumarins, and lignans.

Representative phenolic compounds are presented in Table 5. The addition of ethanol as a co-solvent was associated with a higher relative contribution of several phenolic metabolites, particularly naringenin, quercetin, kaempferol, p-coumaric acid, syringic acid, caffeic acid, apigenin, and resveratrol. Several of these compounds were exclusively detected in the ethanol-modified extract, suggesting that ethanol favored the presence of these metabolites in the resulting extract profile.

Representative lipophilic compounds are presented in Table 6. Both extraction conditions were dominated by oxylipins and unsaturated fatty acids, with 12,13-EODE, 13-HOTrE, γ-linolenic acid, stearidonic acid, and monoolein representing the most abundant tentatively annotated metabolites. These findings are consistent with the non-polar nature of supercritical CO_2_ and its preferential affinity for hydrophobic constituents. The occurrence of triterpenoid-related compounds such as betulin and corosolic acid further demonstrates the ability of supercritical fluid extraction to recover bioactive lipophilic metabolites from grape marc.

Overall, both extraction conditions produced chemically complex extracts containing phenolic and lipophilic metabolites. However, the addition of ethanol broadened the diversity and relative abundance of phenolic constituents while maintaining the extraction of the major lipophilic compounds. These findings provide additional chemical evidence that ethanol modifies extraction selectivity without fundamentally altering the overall chemical profile of the extracts, supporting the trends observed for total phenolic content and antioxidant capacity.

## 3. Discussion

The extraction of bioactive compounds from grape pomace using supercritical CO_2_ is governed by the combined influence of solvent density, solvent polarity, and mass transfer within the solid matrix. Under the optimized conditions identified in this study (10% ethanol, 601 µm, 60 °C, 275.8 bar), the simultaneous improvement of extraction yield, total polyphenol content (TPC), and antioxidant capacity (AC) reflects the need to enhance both solvent strength and polarity. This is consistent with the general behavior of supercritical systems, where pressure increases CO_2_ density and solvent power, while the presence of a co-solvent such as ethanol improves the solubilization of polar compounds, particularly phenolics, which show limited solubility in neat CO_2_ [11,17].

A comparison with previous studies on *Vitis labrusca* pomace shows similar trends, especially regarding the role of ethanol. Co-solvent addition has been consistently associated with improved phenolic recovery, with reported yields around 6–7% and TPC values above 100 mg GAE/g extract at ethanol concentrations between 10–15% [10,11]. These values are in line with those obtained here, supporting the idea that solvent polarity plays a central role in phenolic extraction. From a mechanistic point of view, ethanol not only increases solvent polarity, but may also promote matrix swelling and reduce solute–matrix interactions, which facilitates the release and transport of phenolic compounds into the supercritical phase [10,11]. This interpretation is further supported by the UHPLC-QTOF-MS/MS profiling results, which revealed higher relative abundances of several flavonoids, stilbenes, and phenolic acids, including naringenin, quercetin, p-coumaric acid, caffeic acid, syringic acid, and resveratrol, in the ethanol-modified extracts.

At the same time, the results obtained in this work point to the existence of a selective extraction regime under neat CO_2_ conditions. At 275.8 bar, 30 °C, and 601 µm, neat CO_2_ allowed the recovery of extracts with relatively high TPC (69.3 mg GAE/g) and the highest antioxidant capacity (365.3 µmol TE/g), although the extraction yield remained low (~2%). This behavior highlights the inherent selectivity of CO_2_ toward less polar fractions and suggests that neat CO_2_ operation can be used to obtain extracts with high antioxidant activity and a more selective recovery of target metabolites [14]. Although the extraction yield was lower, the resulting extracts exhibited substantially higher antioxidant capacity and a distinct chemical profile, indicating potential applications where selectivity is prioritized over bulk recovery. This observation is consistent with the chemical profiling results, which revealed a predominance of oxylipins and unsaturated fatty acids in neat CO_2_ extracts despite the presence of detectable phenolic metabolites.

The contrast between selectivity and extraction efficiency becomes clearer when considering additional experiments performed at higher pressures under neat CO_2_ conditions. Increasing pressure from 276 bar to 400 and 500 bar at 30 °C resulted in yields increasing from 1.9% (90 min) to 3.6% and 4.0% within a shorter extraction time (40 min). This behavior is consistent with the increase in solvent density and solvent strength. Even so, the yields remain below those obtained with co-solvent addition, indicating that density alone is not sufficient to compensate for the low polarity of CO_2_ when targeting phenolic compounds. This supports a mechanistic interpretation in which pressure primarily affects solvent strength, while ethanol plays a more decisive role by modifying polarity [16].

From a mass transfer perspective, these trends can be interpreted in terms of the balance between internal and external resistances. Under neat CO_2_ conditions, weaker solute–solvent interactions may increase mass transfer limitations, contributing to lower extraction rates. In contrast, the presence of a co-solvent improves solute solubility and facilitates desorption, enhancing overall mass transfer and extraction performance. This helps explain the differences observed in extraction kinetics.

Particle size also contributed to the observed extraction behavior. Although smaller particles are generally expected to enhance extraction by reducing internal diffusion distances and increasing interfacial area, the results obtained in this study showed improved performance at the upper end of the particle size range. This behavior may be associated with bed structure effects, since larger particles can reduce bed compaction and facilitate solvent flow through the extraction vessel, thereby improving solvent accessibility to the matrix. In contrast, very fine particles may increase flow resistance and promote local channeling or preferential flow paths, partially offsetting the benefits associated with shorter diffusion distances. These observations suggest that, within the experimental domain evaluated, hydrodynamic effects may have played a relevant role alongside mass transfer considerations.

The role of matrix composition becomes more evident when comparing these results with previous studies. Ghafoor et al. reported that pressure and temperature significantly influence extraction from grape peel, while ethanol showed no significant effect within a narrower range (5–8%) [12]. In the present study, ethanol concentration shows a clear effect, which can be attributed to the broader experimental domain and to the use of grape pomace, a more complex and heterogeneous matrix that includes both skins and seeds.

From a process standpoint, the results obtained here are in line with sequential extraction strategies reported in the literature, where the use of co-solvents improves phenolic recovery compared to neat CO_2_ [10]. Reported yields typically range from 4.0% to 7.0%, with TPC values between 70 and 105 mg GAE/g extract at pressures of 300–320 bar, temperatures of 50–60 °C, extraction times of 60–120 min, and ethanol concentrations between 5% and 15%, often using smaller particle sizes (≈300–500 µm). Although solvent-to-feed ratios are not always explicitly reported, the conditions suggest values comparable to those used in this study. Under similar conditions, neat CO_2_ extraction in this work (276 bar, 30 °C, S/F = 0.30 min^−1^, 90 min) yielded 1.9% extract with ~69.3 mg GAE/g, while the optimized co-solvent condition (275.8 bar, 60 °C, 10% ethanol) reached 8.3% yield and ~66.0 mg GAE/g. These results follow the same general trend: neat CO_2_ favors selectivity at low yield, whereas ethanol enhances overall recovery. It is worth noting that comparable yields were obtained despite using larger particle sizes (601–770 µm) and lower ethanol content, suggesting that process performance can be maintained under less restrictive conditions.

The selective neat CO_2_ regime identified at laboratory scale was also reproduced at pilot scale, which supports the robustness of the process. Even with an increase in particle size from 601 to 770 µm, the higher solvent-to-feed ratio (0.36 min^−1^ compared to 0.30 min^−1^ at laboratory scale) resulted in a yield of 2.9 wt% under neat CO_2_ conditions at 30 °C and 276 bar. The extraction kinetics showed the same general behavior observed at laboratory scale, and the slight increase in yield may be associated with differences in solvent-to-feed ratio and extraction bed characteristics between scales. Although hydrodynamic effects could contribute to these differences, additional studies would be required to confirm their specific influence. These results nevertheless demonstrate that the extraction performance observed at laboratory scale can be reproduced under pilot-scale conditions.

Taken together, the results show that supercritical extraction of bioactive compounds from *Vitis labrusca* grape marc can be adjusted to operate under two distinct regimes: a high-recovery regime when a co-solvent is used, and a high-selectivity regime under neat CO_2_ conditions. In line with previous studies, the presence of a co-solvent enhances extraction yield and phenolic recovery, while neat CO_2_ operation favors selectivity. A key contribution of this work is that both regimes can be achieved within a single-step process, with the selective neat CO_2_ regime also validated at the pilot scale. This provides a flexible basis for tailoring extract composition depending on process requirements. UHPLC-QTOF-MS/MS profiling further demonstrated that ethanol-modified extraction was associated with a higher relative abundance and diversity of flavonoids, stilbenes, and phenolic acids, whereas neat CO_2_ extraction favors lipophilic metabolites such as oxylipins and unsaturated fatty acids. These findings support the valorization of grape agro-industrial residues through the production of extracts with distinct chemical profiles and potential applications.

## 4. Materials and Methods

### 4.1. Materials

Grape marc was supplied by the local wine company Casa Domecq (Cali, Colombia). Before processing, the raw material was filtered to remove residual liquid and then oven-dried at a low temperature (below 40 °C) until a constant weight was reached. The dried material was subsequently cryogenically milled and sieved to obtain particle size fractions consistent with the experimental design. After drying, the moisture content was below 5 wt%.

Carbon dioxide (99.9 vol%) and nitrogen (99.99 vol%) were provided by Air Products Cryogas S.A.S. (Cali, Colombia). Liquid nitrogen was obtained from OxiCali Ltda (Cali, Colombia), and anhydrous ethanol was purchased from Inquimicol S.A.S. (Bogotá, Colombia). All reagents were used as received, without any additional purification steps.

### 4.2. Extract Characterization

#### 4.2.1. Overall Extraction Yield

The overall extraction yield (Y) was calculated as the ratio between the mass of extract recovered and the initial mass of dry raw material loaded into the extraction vessel. Results are expressed as weight percentage (wt%).

#### 4.2.2. Total Phenolic Content

Total phenolic content (TPC) was determined using the Folin–Ciocalteu colorimetric method according to Singleton and Rossi [19], with minor adaptations previously reported in the literature [20]. Results are expressed as milligrams of gallic acid equivalents per gram of extract (mg GAE/g). All measurements were performed in triplicate.

#### 4.2.3. Antioxidant Capacity

Antioxidant capacity (AC) was evaluated using the oxygen radical absorbance capacity (ORAC) assay following the methodology proposed by Prior et al. [21], with minor adaptations described elsewhere [20]. Results are expressed as micromoles of Trolox equivalents per gram of extract (µmol TE/g). All measurements were performed in triplicate.

#### 4.2.4. UHPLC-QTOF-MS/MS Analysis of Representative Extracts

Representative extracts obtained under neat CO_2_ and ethanol-modified CO_2_ conditions were further characterized by ultra-high-performance liquid chromatography coupled to quadrupole time-of-flight tandem mass spectrometry (UHPLC-QTOF-MS/MS). Extract samples were dissolved in a methanol–water solution (50:50, *v*/*v*), vortex-mixed for 1 min, sonicated for 5 min, filtered through 0.22 µm hydrophilic PTFE filters, and transferred to chromatographic vials for analysis.

Chromatographic analyses were performed using a Bruker Elute UHPLC system coupled to a Bruker Impact II high-resolution QTOF mass spectrometer (Bruker Daltonics, Bremen, Germany). Separation was carried out on a Bruker Intensity Solo 2 C18 column (100 × 2.1 mm) maintained at 30 °C. The mobile phase consisted of (A) water containing 0.1% formic acid and (B) acetonitrile containing 0.1% formic acid. Elution was performed using the following gradient program (%B): 5/5/95/95/5/5 at 0/1/11/17/17.1/20 min. The flow rate was 0.25 mL min^−1^ and the injection volume was 5 µL.

Mass spectrometric analyses were performed using an electrospray ionization (ESI) source operating in both positive and negative ionization modes. Data were acquired in Auto MS/MS mode over a mass range of *m*/*z* 70–2000. The source parameters were as follows: capillary voltage, 4500 V; end plate offset, 500 V; nebulizer pressure, 29 psi; dry gas flow, 9.0 L min^−1^; and dry gas temperature, 200 °C.

Data processing and compound annotation were performed using Bruker Compass MetaboScape software (version 6.0.2, Bruker Daltonics, Bremen, Germany). Compound annotation was based on accurate mass measurements, isotopic patterns, fragmentation spectra, and database matching using Bruker MetaboBASE Personal Library 3.0, MoNA MassBank, MassBank Europe, GNPS, and LipidBlast libraries. The resulting annotations were subsequently subjected to manual curation to retain only chemically plausible metabolites relevant to grape-derived matrices. Relative abundances were calculated from normalized peak areas and are reported as relative signal contributions rather than absolute concentrations. Accordingly, all reported metabolites should be considered as tentatively annotated compounds based on high-resolution mass spectrometric evidence and database matching rather than unequivocally identified compounds. The complete list of tentatively annotated compounds retained after manual curation is provided in Supplementary Table S1.

### 4.3. Laboratory-Scale Supercritical Fluid Extraction Procedure

**Figure 5:**
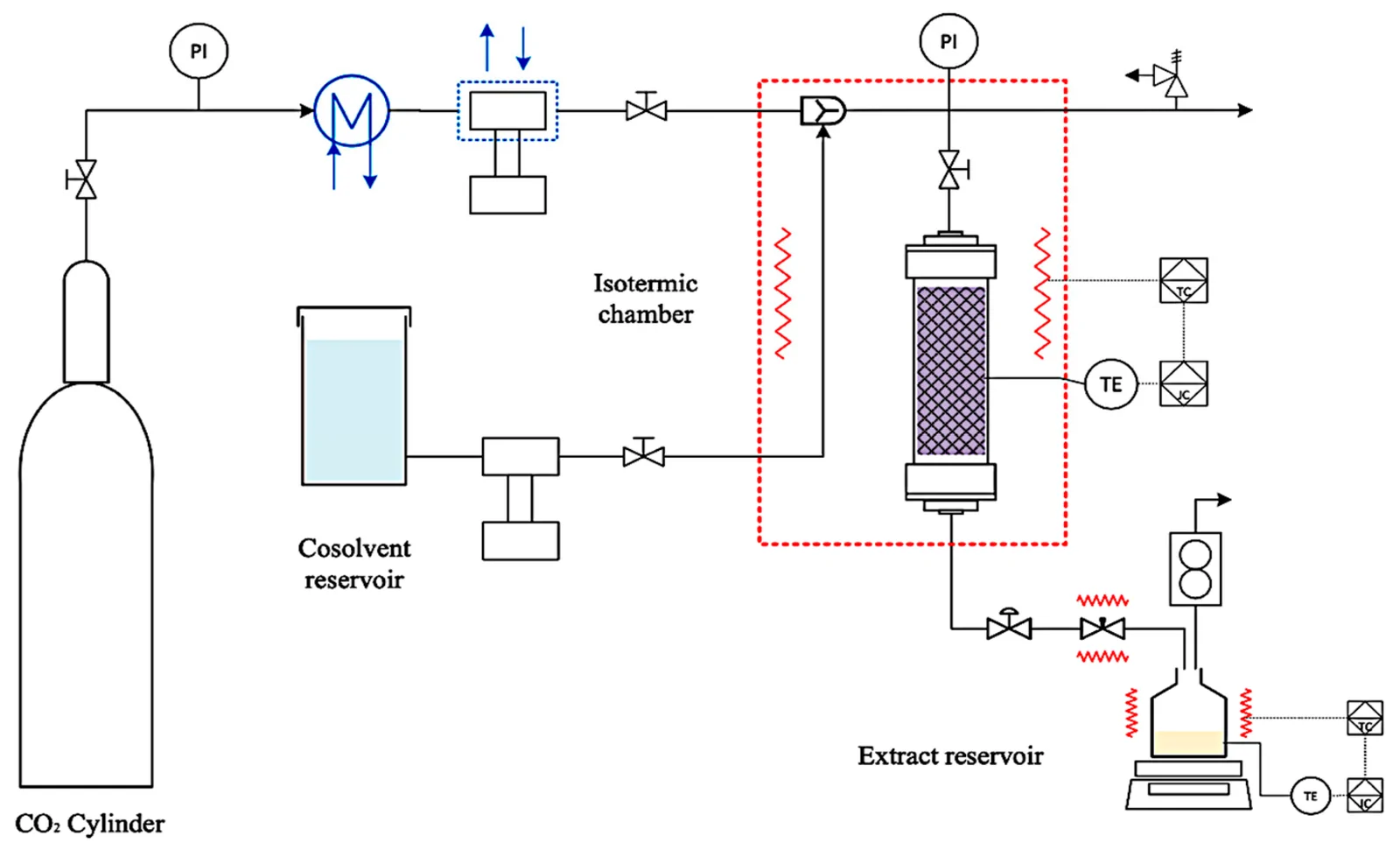
Schematic diagram of the laboratory-scale supercritical fluid extraction (SFE) system used for the recovery of bioactive compounds from *Vitis labrusca* grape marc. Red dashed lines indicate the isothermal chamber enclosing the extraction vessel. PI denotes pressure indicators, and TE denotes temperature sensors.

Figure 5 shows a schematic representation of the laboratory-scale supercritical fluid extraction (SFE) system used in this work. The setup is organized into three main sections: CO_2_ supply and pumping, extraction, and separation.

Carbon dioxide is delivered from a storage cylinder and cooled below 5 °C using a chiller to maintain it in the liquid phase before pumping. The fluid is then driven by a high-pressure pump (Ligao Pump Co., Ltd., Wuxi, China, model JSZ6/40) toward the extraction section. System pressure is controlled by restricting the flow with a manual micrometric valve located downstream of the extractor, allowing stable operation at the desired pressure. Before entering the extraction vessel, CO_2_ is combined with ethanol in a static mixer. The co-solvent is introduced using an HPLC pump (JASCO Corporation, Tokyo, Japan, model PU-980) from a separate reservoir, ensuring a controlled and continuous feed. The resulting CO_2_–ethanol mixture passes through the extractor, which is packed with grape marc and housed inside an isothermal chamber to maintain uniform temperature conditions. Heating elements and temperature sensors (TE) are used to regulate the operating temperature.

After extraction, the fluid stream leaves the vessel and flows through the micrometric valve, where depressurization takes place. This pressure drop promotes the precipitation of the extracted compounds in the collection vessel, while CO_2_ is vented through the outlet line. The extract is collected in a flask under oxygen-free conditions. Pressure is monitored using pressure indicators (PI), and temperature is controlled both in the extraction and separation sections. The CO_2_ flow is continuously adjusted to ensure stable operation throughout the process.

For each experimental run, approximately 50 g of dried grape marc was loaded into the extraction vessel. The extraction experiments were carried out using a CO_2_ flow rate of 15 g min^−1^, which was maintained constant throughout the extraction period. The extraction time was fixed at 90 min for all experiments. When required by the experimental design, ethanol was continuously introduced as a co-solvent using the HPLC pump and expressed as a weight percentage relative to the total solvent flow.

### 4.4. Experimental Design and Data Analysis

A 2^4−1^ fractional factorial design was used to examine the effect of four independent variables—temperature (T), pressure (P), ethanol concentration as co-solvent (C), and average particle size (S)—on three response variables: antioxidant capacity (AC), total phenolic content (TPC), and overall extraction yield (Y). The selected configuration corresponds to a Resolution IV design, which allows the estimation of main effects without confounding with other main effects or two-factor interactions, under the assumption that higher-order interactions are negligible within the studied experimental domain. This type of design is commonly applied as a first approach to identify the most relevant variables while keeping the number of experimental runs manageable.

The experimental factors were defined within a practical operating window, with temperature ranging from 30 to 60 °C, pressure from 137.9 to 275.8 bar, ethanol concentration from 0 to 10 wt%, and particle size from 116 to 601 µm. These variables were coded between −1 and 1 to simplify the statistical analysis and allow direct comparison of their effects. Central points (0, 0, 0, 0) were also included to check for possible curvature in the response surfaces and to gain insight into the reproducibility of the experiments. As with any fractional factorial screening design, quadratic effects were not explicitly estimated, and some higher-order interactions remained confounded. Consequently, the resulting models are intended to identify influential variables and trends within the experimental domain rather than to provide a complete description of the extraction process. This may contribute to the lower adjusted R^2^ observed for the extraction yield. Therefore, these models should be interpreted primarily as screening and trend-identification tools within the studied experimental domain rather than as predictive models intended for extrapolation beyond the evaluated conditions.

The structure of the design was defined by the generator I = CTPS, which determines the aliasing pattern. Under this configuration, each main effect is confounded with a three-factor interaction but remains independent of other main effects and two-factor interactions. In practical terms, the alias structure can be written as C = STP, S = CTP, T = CSP, and P = CST. This arrangement is consistent with Resolution IV designs and relies on the assumption that three-factor interactions are small enough to be neglected, allowing a meaningful interpretation of the main effects.

From a practical perspective, this design offers an efficient way to explore the multidimensional factor space with a reduced number of experiments. At the same time, it retains enough sensitivity to identify the most influential variables and their interactions, which is particularly important in extraction systems where solubility, mass transfer, and matrix–solute interactions occur simultaneously.

The behavior of the response variables was described using empirical models obtained through multiple linear regression. Analysis of variance (ANOVA) was used to evaluate the contribution of each term and to assess the adequacy of the models. During model refinement, terms with *p*-values above 0.05 were removed while preserving model hierarchy whenever possible. In cases where hierarchy could not be strictly maintained, the final model was selected based on a combination of statistical significance, residual analysis, predictive performance, and consistency with the underlying extraction phenomena.

All statistical analyses were carried out using JMP^®^ software (version 14, SAS Institute Inc., Cary, NC, USA). The resulting models were then used to interpret the response behavior across the experimental domain and to identify suitable operating conditions through a desirability-based approach. This strategy makes it possible to consider all responses simultaneously, providing a more balanced basis for process optimization and subsequent scale-up.

### 4.5. Pilot-Scale Supercritical Fluid Extraction System

A pilot-scale supercritical fluid extraction (SFE) unit was developed and assembled by Didacontrol S.A.S., taking as reference the operating conditions identified at laboratory scale. The system includes two extraction vessels, each with an internal volume of 10 L and a height-to-diameter (H/D) ratio of 5. These extractors are connected to two separators with a capacity of 3 L each, allowing operation either in series or in parallel, depending on the process requirements.

During scale-up, key operating parameters were preserved as much as possible, including an apparent feed density of 0.3 kg·L^−1^ and a solvent-to-feed ratio in the range of 0.4–0.5 min^−1^. Under these conditions, the system operates with a CO_2_ flow rate close to 180 kg·h^−1^, which ensures sufficient solvent availability throughout the extraction process.

The unit can operate at pressures up to 440 bar and temperatures up to 100 °C, and it was built using stainless steel (SS 316/304) to ensure both mechanical resistance and compatibility with supercritical CO_2_ and co-solvents. Operation is supported by a semi-automatic control panel, which allows continuous monitoring of pressure, temperature, and flow conditions, contributing to stable and reproducible performance. Before starting the experimental campaigns, the system was subjected to safety and performance checks to verify its integrity and proper operation.

## 5. Conclusions

This study demonstrated that supercritical CO_2_ extraction of bioactive compounds from *Vitis labrusca* grape marc can be tuned through operating conditions to achieve two distinct extraction regimes. Under ethanol-modified conditions (275.8 bar, 60 °C, 10 wt% ethanol, 601 µm, S/F = 0.30 min^−1^, 90 min), the process favored overall recovery, reaching an extraction yield of 8.3 wt%, together with a total phenolic content (TPC) of approximately 66.0 mg GAE/g and an antioxidant capacity (AC) of 250.2 µmol TE/g. In contrast, neat CO_2_ extraction (276 bar, 30 °C, 601 µm, S/F = 0.30 min^−1^, 90 min) produced lower extraction yields (1.9 wt%) but extracts with higher antioxidant capacity (365.3 µmol TE/g) and comparable TPC values (69.3 mg GAE/g), indicating a more selective extraction regime.

Additional experiments performed under neat CO_2_ at higher pressures (400–500 bar) confirmed that increasing solvent density enhances extraction yield; however, the results also showed that density alone cannot compensate for the limited polarity of CO_2_ when targeting phenolic compounds. These findings highlight the complementary roles of solvent density and solvent polarity in governing extraction performance.

The UHPLC-QTOF-MS/MS characterization provided additional chemical insight into the differences between extraction regimes. Ethanol-modified extraction was associated with a higher relative abundance and diversity of flavonoids, stilbenes, and phenolic acids, whereas neat CO_2_ extraction favored lipophilic metabolites, particularly oxylipins and unsaturated fatty acids. These results confirm that ethanol not only improves extraction yield but also modifies extraction selectivity, resulting in extracts characterized by a higher relative representation of polar bioactive compounds.

The use of a 2^4−1^ fractional factorial design enabled the identification of the most influential process variables while providing a practical framework for process optimization. Furthermore, the selective neat CO_2_ regime was successfully reproduced at pilot scale, reaching extraction yields of 2.9 wt% under comparable operating conditions, thereby supporting the scalability of the proposed approach.

Overall, the results demonstrate that supercritical CO_2_ extraction can be tuned to produce extracts with distinct compositional and functional characteristics by adjusting operating conditions and co-solvent addition. The ability to switch between a high-recovery regime and a high-selectivity regime provides a versatile platform for tailoring grape marc extracts according to specific industrial requirements. From a process engineering perspective, this flexibility enables the design of extraction strategies that prioritize either product yield or extract selectivity, thereby facilitating the industrial implementation of supercritical fluid technologies for the valorization of winery by-products.

## Figures and Tables

**Table 1:** Results of the 2^4−1^ fractional factorial experimental design proposed.

T	P	C	S	%Y (90 min)	TPC (mg GAE/g)			AC (μmol TE/g)		
1	−1	1	−1	7.1	64.0	±	1.7	238.4	±	3.7
0	0	0	0	3.8	36.9	±	1.5	146.4	±	3.3
−1	−1	−1	−1	1.2	6.3	±	0.1	40.8	±	0.8
0	0	0	0	2.7	44.0	±	1.7	178.6	±	2.3
−1	−1	1	1	5.8	21.8	±	0.6	95.4	±	1.5
−1	1	−1	1	1.9	69.3	±	0.9	365.3	±	2.4
1	−1	−1	1	0.3	23.8	±	0.5	177.4	±	1.4
0	0	0	0	2.0	37.0	±	1.4	160.8	±	1.7
−1	1	1	−1	2.2	44.1	±	0.9	175.6	±	1.9
1	1	−1	−1	1.4	21.9	±	0.6	139.6	±	3.5
1	1	1	1	5.2	65.6	±	0.9	244.6	±	2.9

Where coded variables T: temperature [30, 60] °C, P: pressure [137.9, 275.8] bar, C: ethanol concentration [0, 10] wt.%, S: average particle size [116, 601] μm, %Y: extraction yield (SD = 0.9%), TPC: total polyphenol content, GAE: Gallic Acid Equivalent, AC: antioxidant capacity, TE: Trolox Equivalent.

**Table 2:** *p*-values of the main factor and selected binary interactions.

Factor	*p*-Value
C × T	0.00217
C × S	0.00282
S	0.00288
T	0.00427
P	0.01835
C × P	0.02903
C	0.03716

**Table 3:** *p*-values of the main factor and selected binary interactions after refinement.

Factor	*p*-Value
C × T	0.01074
C × S	0.01356
S	0.01364
T	0.01560
P	0.05687

**Table 4:** Estimated regression coefficients of the fitted models for extraction yield, total polyphenol content, and antioxidant capacity.

Term	TPC (β ± SE)	*p*-Value	AC (β ± SE)	*p*-Value	Ln(Y) (β ± SE)	*p*-Value
β_0_	39.52 ± 2.52	<0.0001	178.45 ± 12.47	<0.0001	0.830 ± 0.177	0.0054
β_4_ (S)	10.63 ± 2.95	0.0156	46.64 ± 14.62	0.0243	0.090 ± 0.208	0.6836
β_1_ (T)	9.28 ± 2.95	0.0257	3.86 ± 14.62	0.8022	0.775 ± 0.208	0.0136
β_2_ (P)	5.53 ± 2.95	0.1204	36.04 ± 14.62	0.0569	−0.053 ± 0.208	0.8095
β_3_ (C) × β_4_ (S)	−10.70 ± 2.95	0.0152	−54.54 ± 14.62	0.0136	0.217 ± 0.208	0.3438
β_3_ (C) × β_1_ (T)	11.70 ± 2.95	0.0107	37.64 ± 14.62	0.0498	0.344 ± 0.208	0.1588

SE: standard error. Coefficients are expressed in coded variables.

**Table 5:** Representative phenolic compounds tentatively annotated by UHPLC-QTOF-MS/MS in extracts obtained with neat CO_2_ and ethanol-modified CO_2_.

Chemical Class/Subclass	Compound	Relative Abundance (%)		Fold Change (EtOH/CO_2_)
		SCFE CO_2_	SCFE + EtOH	
Flavanones	Naringenin	0.011	0.512	46.5
Flavonols	Quercetin	0.009	0.241	26.8
	Kaempferol	0.001	0.027	27.0
	Kaempferol-4′-methyl ether	ND	0.013	Only in EtOH
Flavones	Apigenin	ND	0.091	Only in EtOH
Stilbenes	Resveratrol	ND	0.040	Only in EtOH
	Resveratrol 4′-methyl ether	0.002	0.013	6.5
Hydroxybenzoic acids	Syringic acid	0.083	0.331	4.0
Hydroxycinnamic acids	p-Coumaric acid	0.012	0.202	16.8
	trans-Caffeic acid	ND	0.129	Only in EtOH
	trans-Ferulic acid	0.014	0.031	2.2

ND = not detected. Relative abundances were calculated from normalized UHPLC-QTOF-MS/MS peak areas and should be interpreted as relative signal contributions rather than absolute concentrations. Compound identities correspond to tentative annotations based on accurate mass and MS/MS fragmentation data.

**Table 6:** Representative lipophilic compounds tentatively annotated by UHPLC-QTOF-MS/MS in extracts obtained with neat CO_2_ and ethanol-modified CO_2_.

Chemical Class/Subclass	Compound	Relative Abundance (%)		Fold Change (EtOH/CO_2_)
		SCFE CO_2_	SCFE + EtOH	
Oxylipins	12,13-EODE	24.547	22.777	0.93
	13-HOTrE	19.791	13.399	0.68
	13-OxoODE	ND	5.714	Only in EtOH
Unsaturated fatty acids	γ-Linolenic acid	13.411	14.264	1.06
	Stearidonic acid	4.120	4.177	1.01
Fatty acid derivatives	Stearidonic acid methyl ester	0.027	0.031	1.15
Monoacylglycerols	Monoolein	4.134	4.448	1.08
Triterpenoids	Corosolic acid	0.067	0.109	1.63
	Betulin	0.043	0.171	3.98

ND = not detected. Relative abundances were calculated from normalized UHPLC-QTOF-MS/MS peak areas and should be interpreted as relative signal contributions rather than absolute concentrations. Compound identities correspond to tentative annotations based on accurate mass and MS/MS fragmentation data.

## Data Availability

The original contributions presented in this study are included in the article/supplementary material. Further inquiries can be directed to the corresponding author(s).

## Supplementary Materials

The following supporting information can be downloaded at https://www.mdpi.com/article/10.3390/molecules31132272/s1: Figure S1. Residual diagnostic analysis of the fitted models for (a) total phenolic content (TPC), (b) antioxidant capacity (AC), and (c) extraction yield (ln(Y)), including normal probability plots of residuals and residuals versus predicted values. Figure S2. Comparative extraction kinetics for (a) pure CO_2_ and (b) CO_2_ with ethanol as co-solvent, highlighting the influence of solvent polarity and density on extraction rate, mass transfer dynamics, and the trade-off between selectivity and overall recovery. Table S1. Curated list of tentatively annotated compounds retained for interpretation after UHPLC-QTOF-MS/MS profiling of representative extracts obtained with neat CO_2_ and ethanol-modified CO_2_.
