# Supplementary material for: Supercritical CO2 Extraction of Bioactive Compounds from Vitis labrusca Grape Marc: Effects of Operating Conditions and Pilot-Scale Validation
Source: Molecules. 2026 Jun 29;31(13):2272. doi: 10.3390/molecules31132272 (PMC13362750; doi:10.3390/molecules31132272)
Supplement: Supplementary file 1 [file molecules-31-02272-s001.zip › molecules-4292061-supplementary.pdf]

<u>Supplementary material</u>

Supercritical CO_2 Extraction of Bioactive Compunds from *Vitis labrusca* Grape Marc: Effects of Operating Conditions and Pilot-scale Validation

Camilo Pardo-Castaño [1,2,*], Alejandro Quintero-Velez [1,2] and William Fernando Vallejo-Revelo [2]

[1] School of Chemical Engineering, Universidad del Valle, Calle 13 No. 100-00, Cali 760032, Colombia
[2] Didacontrol S.A.S., Calle 32 No. 1-22, Cali 760001, Colombia
[*] Correspondence: camilo.pardo@correounivalle.edu.co

Residual diagnostic

Figure S1 shows the Residual diagnostic plots that were used to assess the adequacy of the fitted models for total phenolic content (TPC), antioxidant capacity (AC), and extraction yield (ln(Y)). The analysis includes normal probability plots of residuals and residuals versus predicted values.

The residuals exhibited an approximately normal distribution, with no significant deviations from linearity observed in the normal probability plots. Additionally, the residuals versus predicted values showed no discernible patterns, indicating homoscedasticity across the experimental domain. No systematic trends were observed in relation to run order, confirming the independence of observations.

These results support the validity of the fitted models and indicate that the underlying assumptions of linear regression are reasonably satisfied within the experimental domain.

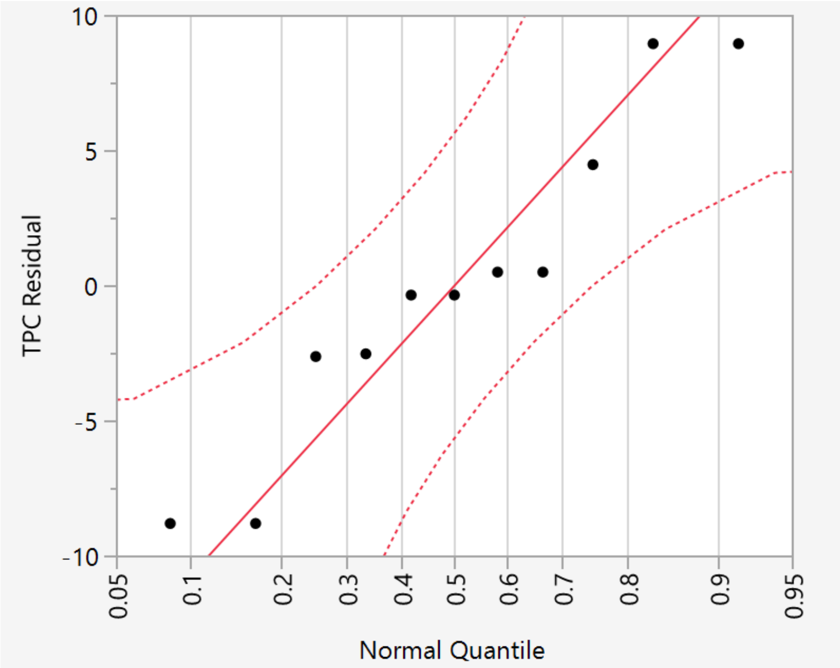

TPC Residual
Normal Quantile
10
5
0
-5
-10
0.05
0.1
0.2
0.3
0.4
0.5
0.6
0.7
0.8
0.9
0.95

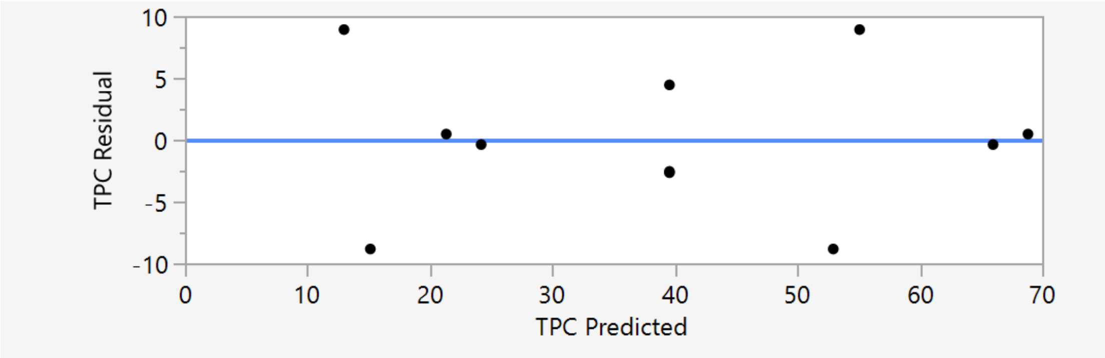

TPC Residual
TPC Predicted
10
5
0
-5
-10
0
10
20
30
40
50
60
70

(a)

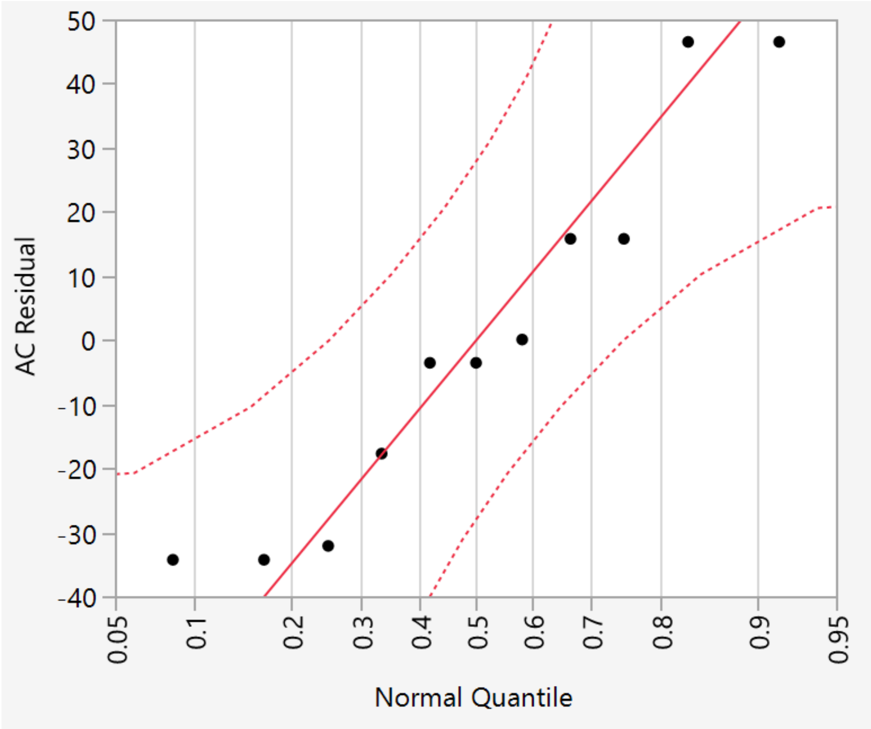

AC Residual
50
40
30
20
10
0
-10
-20
-30
-40
0.05
0.1
0.2
0.3
0.4
0.5
0.6
0.7
0.8
0.9
0.95
Normal Quantile

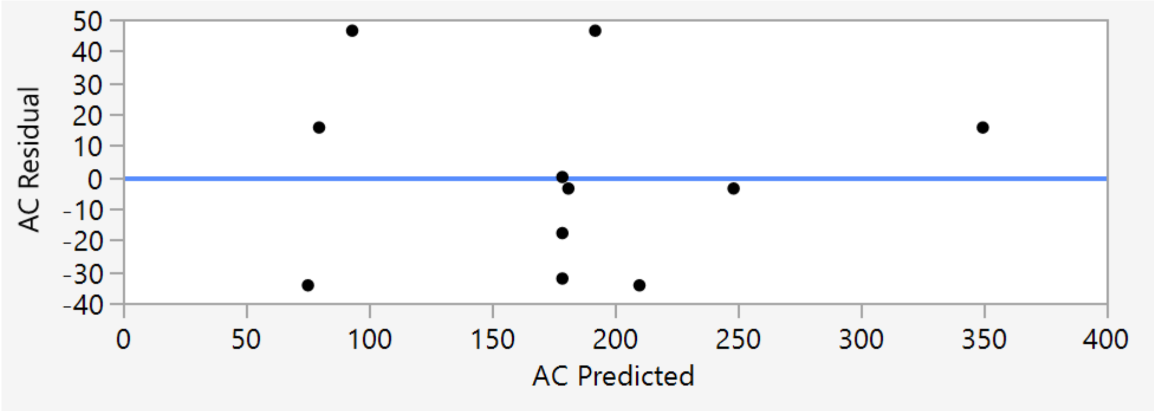

AC Residual
50
40
30
20
10
0
-10
-20
-30
-40
0
50
100
150
200
250
300
350
400
AC Predicted

(b)

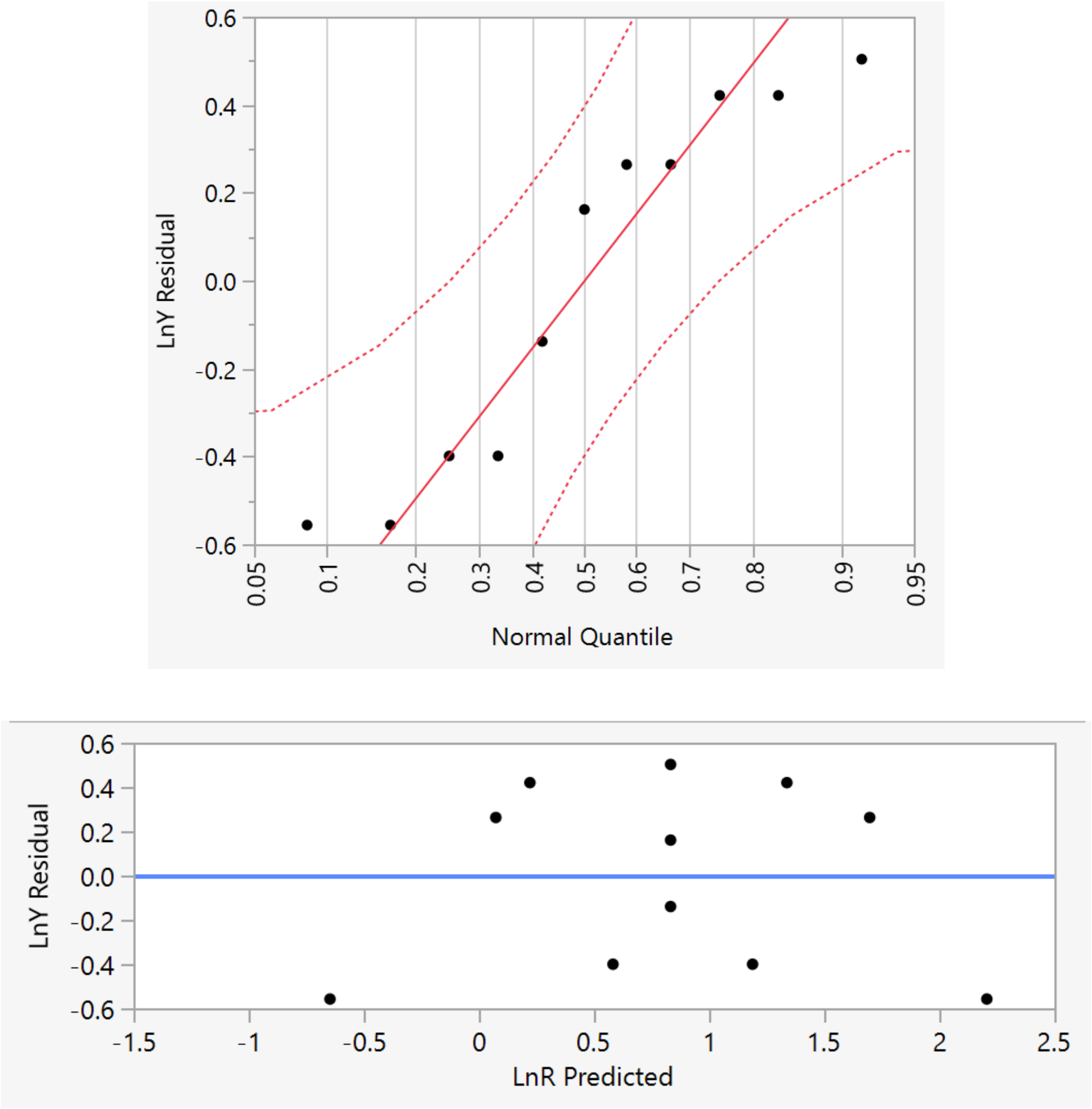

(c)

Figure S1. Residual diagnostic analysis of the fitted models for (a) total phenolic content (TPC), (b) antioxidant capacity (AC), and (c) extraction yield (ln(Y)), including normal probability plots of residuals and residuals versus predicted values.

Extraction kinetics

Additional extraction kinetics were analyzed to further evaluate the influence of operating conditions on extraction performance. The results confirm that increasing pressure enhances

extraction rates under solvent-free conditions due to increased CO_2 density, while the addition of ethanol significantly improves extraction efficiency by increasing solvent polarity.

The presence of co-solvent results in faster initial extraction rates and higher overall recovery, suggesting enhanced solute desorption and improved mass transfer within the solid matrix. These observations are consistent with the trends discussed in the main manuscript and further support the identified trade-off between extraction yield and selectivity.

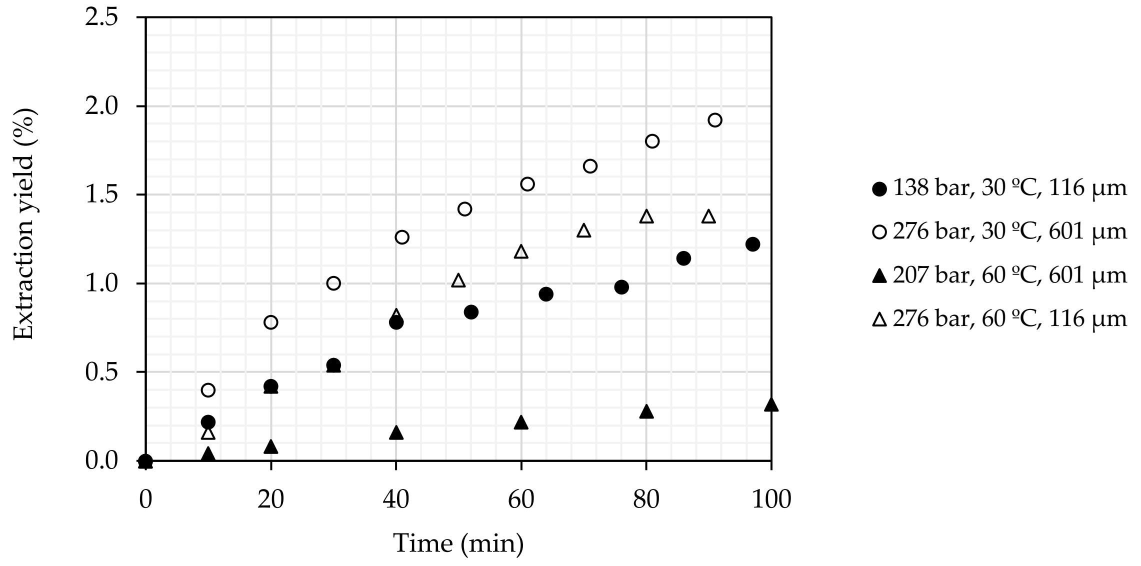

(a)

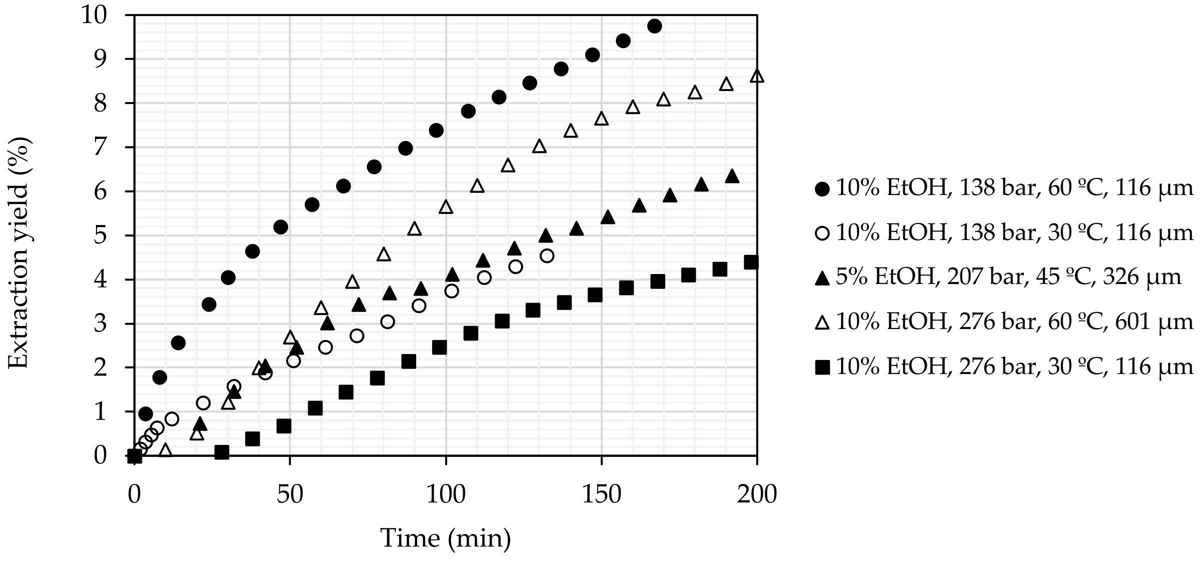

(b)

Figure S2. Comparative extraction kinetics for (a) pure CO_2 and (b) CO_2 with ethanol as co-solvent, highlighting the influence of solvent polarity and density on extraction rate, mass transfer dynamics, and the trade-off between selectivity and overall recovery.

Chemical Profiling

Table S1. Curated list of tentatively annotated compounds retained for interpretation after UHPLC-QTOF-MS/MS profiling of representative extracts obtained with neat CO_2 and ethanol-modified CO_2.

Chemical Class/Subclass	Compound	Relative abundance SCFE CO_2 (%)	Relative abundance SCFE+EtOH (%)
**Oxylipins**	12,13-EODE	24.547	22.777
	13-HOTrE	19.791	13.399
	13-OxoODE	ND	5.714
	9-HODE	2.789	2.167
	(±)12,13-DiHOME	2.249	2.153
	9(S)-HOTrE	2.126	1.496
	trans-EKODE-(E)-Ib	1.520	1.244
	19S-HETE	1.151	0.829
	(±)11,12-DHET	0.668	0.460
	17(S)-HETE	0.535	0.404
	15(S)-HEDE	0.196	0.160
	9-KODE	0.021	0.028
	9(S)-HpODE	0.009	0.008
	(±)5(6)-EET methyl ester	0.015	0.008
**Oxylipins / Eicosanoids**	9,11-methane-epoxy PGF1α	1.107	0.967
	11-deoxy-PGF1α	0.282	0.553
	PGF1α	0.156	0.104

Chemical Class/Subclass	Compound	Relative abundance SCFE CO_2 (%)	Relative abundance SCFE+EtOH (%)
**Unsaturated fatty acids**	γ-Linolenic acid	13.411	14.264
	Stearidonic acid	4.120	4.177
	9,12-Octadecadiynoic acid	3.856	3.012
	ω-3 Arachidonic acid	0.438	0.644
	Linolenic acid ethyl ester	0.394	0.852
	ω-3 Arachidonic acid ethyl ester	0.110	0.148
	Stearidonic acid methyl ester	0.027	0.031
**Hydroxy fatty acids**	2-hydroxyhexadecanoic acid	1.895	2.419
	(9Z,12R)-12-Hydroxyoctadec-9-enoic acid	0.627	0.563
	(R)-2-hydroxystearic acid	0.337	0.874
	12-Hydroxydodecanoic acid	0.187	0.159
	2-Hydroxymyristic acid	0.081	0.014
**Fatty-acid hydroxy esters**	9-OAHSA	0.029	0.062
**Dicarboxylic acids**	Azelaic acid	0.060	0.330
	Tridecanedioic acid	0.155	0.171
	Dodecanedioic acid	0.042	0.160

Chemical Class/Subclass	Compound	Relative abundance SCFE CO_2 (%)	Relative abundance SCFE+EtOH (%)
	Undecanedioic acid	0.028	0.122
	Octadecanedioic acid	0.120	0.091
	4-Oxododecanedioic acid	0.009	0.078
	Sebacic acid	0.014	0.042
**Monoacylglycerols**	Monoolein	4.134	4.448
	MG(18:2)/0:0/0:0	2.245	1.971
**Monoacylglycerol ethers**	2-AG ether	0.061	0.266
**Lipid amides**	N-Oleoylethanolamine	0.460	0.571
**Amino lipids**	2-aminohexadecanoic acid	ND	0.087
**Sphingolipids**	D-sphingosine	0.297	0.439
	Sphinganine	0.002	0.633
	3-ketosphinganine	ND	0.307
**Diterpenes**	Incensole	0.767	0.464
	Isosteviol	0.422	0.354
**Triterpenes**	Corosolic acid	0.067	0.109
	Betulin	0.043	0.171
**Terpenoid phytohormones**	Abscisic acid	0.025	0.045
**Fatty-acid-derived phytohormones**	Dihydrojasmonic acid methyl ester	0.039	0.033

Chemical Class/Subclass	Compound	Relative abundance SCFE CO_2 (%)	Relative abundance SCFE+EtOH (%)
**Isoprenoid intermediates**	(±)-Mevalonic acid 5-phosphate	0.039	0.015
**Norisoprenoids**	Dihydroactinidiolide	1.284	0.903
**Flavanones**	Naringenin	0.011	0.512
	Pinocembrin	0.078	0.195
**Methylated flavanones**	Sakuranetin	0.004	0.015
**Flavanonols**	Fustin	0.003	0.018
**Flavones**	Apigenin	ND	0.091
**Methoxylated flavones**	6-Methoxyluteolin	0.020	0.360
**Flavonols**	Quercetin	0.009	0.241
	Kaempferol	0.001	0.027
	Kaempferol-4′-methyl ether	ND	0.013
**Methylated flavonols**	Rhamnetin	0.018	0.090
**Stilbenes**	Resveratrol	ND	0.040
	Resveratrol 4′-methyl ether	0.002	0.013
**Hydroxybenzoic acids**	Syringic acid	0.083	0.331
**Hydroxycinnamic acids**	p-Coumaric acid	0.012	0.202
	trans-Caffeic acid	ND	0.129

Chemical Class/Subclass	Compound	Relative abundance SCFE CO_2 (%)	Relative abundance SCFE+EtOH (%)
	trans-Ferulic acid	0.014	0.031
**Methoxylated phenolic acids**	5-Methoxysalicylic acid	0.088	0.148
**Methoxylated cinnamic acids**	4-Methoxycinnamic acid	0.009	0.016
**Caffeic acid esters**	Phenethyl caffeate	0.020	0.177
**Coumaric acid esters**	Ethyl-p-coumarate	6.390	5.413
**Phenolic esters**	Ethyl syringate	0.144	0.084
**Coumarins**	Scopoletin	0.053	0.103
	6,7-Dihydroxycoumarin	ND	0.088
**Chromones**	7-Methoxychromone	0.031	0.075
**Lignans**	Matairesinol	ND	0.028
**Depsides**	Divaricatic acid	0.023	0.062
